# Transsacral Screws in Combination With an Expandable Cage for High-Grade Spondylolisthesis: A Novel Technique Involving Transsacral/Transforaminal Lumbar Interbody Fusion

**DOI:** 10.7759/cureus.100839

**Published:** 2026-01-05

**Authors:** Evangelos Christodoulou, Theodoros Grivas

**Affiliations:** 1 Clinic for Spine and Pain, St. Vinzenz Hospital, Düsseldorf, DEU; 2 Orthopaedics and Traumatology, Tzaneio General Hospital of Piraeus, Piraeus, GRC

**Keywords:** expandable cage, high-grade spondylolisthesis, slip angle, spinal fusion, transforaminal lumbar interbody fusion, transsacral screws

## Abstract

High-grade spondylolisthesis at the L5/S1 level presents significant surgical challenges due to marked instability and neural compression. Traditional fusion techniques often need to extend beyond the index level to achieve stable fixation. Moreover, a second surgery is sometimes required to restore mobility at the additionally instrumented segments.

We present a case of a 19-year-old female with severe lumbar and bilateral sciatic pain caused by a Meyerding grade III L5/S1 spondylolisthesis. The patient underwent a laminectomy and bilateral L5 nerve root decompression, followed by transsacral screw fixation combined with an expandable interbody cage. Postoperatively, she achieved marked pain relief, restoration of lumbosacral alignment, and radiographic evidence of solid fusion at six months. No neurological deficits or implant-related complications were observed.

This technical note describes a novel single-stage approach for the treatment of high-grade L5/S1 spondylolisthesis. The key advantage of this technique lies in its biomechanical stability, achieved by combining transsacral screw fixation with an interbody cage, unlike previous methods that used either longer-segment instrumentation with a cage or transsacral screws without a cage. These findings are limited by the single-case nature and short follow-up period, but the technique may represent a promising option for enhanced stabilization in high-grade slips.

## Introduction

High-grade spondylolisthesis is a rare but debilitating condition, accounting for approximately 11.3% of all spondylolisthesis cases, and most commonly affects the lumbosacral junction [1]. In adolescents and young adults, it is frequently associated with severe low back and radicular pain, gait disturbances, and progressive deformity, often necessitating surgical intervention.

Most authors agree that spinal fusion is indicated in symptomatic high-grade cases; however, surgical management remains challenging due to the complex interplay of spinal instability, lumbosacral kyphosis, neural compression, and altered spinopelvic alignment [2]. Restoration of sagittal balance is increasingly recognized as a critical goal, as inadequate correction may contribute to persistent symptoms and adjacent segment degeneration.

Traditional surgical strategies include anterior, posterior, or combined fusion techniques, ranging from in situ fusion to partial or complete reduction of the slip [3,4]. In high-grade deformities, longer constructs extending fixation to L4 or the iliac bones are frequently employed to enhance initial stability [5,6]. While effective, such extended instrumentation is associated with increased operative morbidity, greater blood loss, loss of adjacent motion segments, and, in some cases, the need for secondary procedures to remove proximal fixation. For young patients in particular, preservation of adjacent motion segments and avoidance of iliac fixation are important considerations to reduce long-term functional impairment.

Despite the wide range of described techniques, there remains no consensus regarding the optimal balance between achieving adequate stability and minimizing surgical invasiveness. In particular, strategies that provide sufficient anterior and posterior column support while avoiding extended fixation remain underrepresented in the literature.

In this context, we present a modified and less extensive fusion strategy combining transsacral screw fixation with an expandable interbody cage inserted via a transforaminal lumbar interbody fusion (TLIF) approach for L5/S1 stabilization. This technique was applied in a 19-year-old female with Meyerding grade III L5/S1 spondylolisthesis presenting with severe lumbar and bilateral sciatic pain. The proposed approach aims to achieve neural decompression, mechanical stability, correction of slip angle and lumbosacral kyphosis, and restoration of anterior column support, while limiting the extent of fusion and avoiding extension of instrumentation to L4 or the ilium.

## Technical report

Patient presentation

A 19-year-old female presented with progressively worsening lower back pain and bilateral leg pain radiating along the sciatic distribution. She reported a visual analog scale (VAS) score of 5/10 for low back pain and 9/10 for bilateral leg pain. She was 161 cm tall and weighed 50 kg, corresponding to a body mass index (BMI) of 19.3. Neurological examination revealed positive straight-leg raise tests bilaterally, without motor deficits.

Imaging

**Figure 1:**
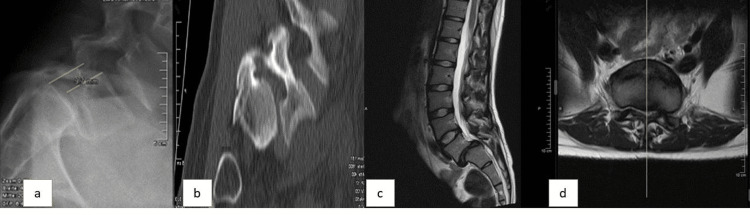
(a) Lateral X-ray with III° spondylolisthesis. (b) Sagittal CT view showing the pars elongation and the dysplastic pedicle. T2 MRI showing the foraminal (c) and central (d) stenosis.

Plain radiographs and magnetic resonance imaging (MRI) confirmed an unstable high-grade spondylolisthesis at L5/S1 (Meyerding grade III, Wiltse-Newman isthmic type IIB), with elongation of the pars interarticularis causing both foraminal and central canal stenosis (Figure 1).

Surgical technique

Positioning

**Figure 2:**
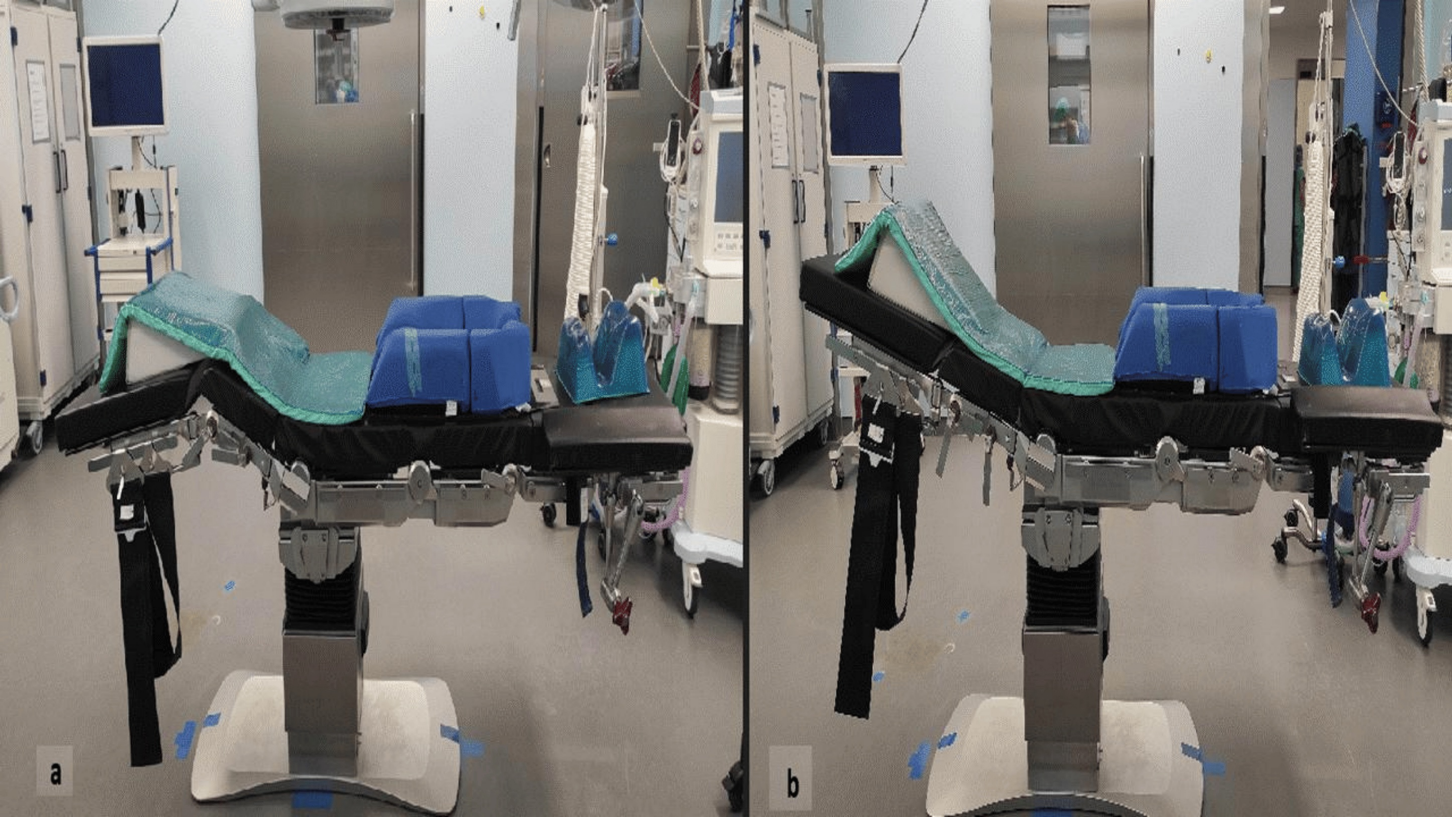
(a) The patient's knees and hips were first flexed for sciatic nerve protection, and (b) extended intraoperatively to achieve reduction.

After induction of general anesthesia and endotracheal intubation, the patient was positioned prone on a Jackson table with a positioning frame, allowing the abdomen to hang freely. The hips and knees were flexed to reduce tension on the sciatic nerve (Figure 2A). A standard posterior midline approach was performed through an approximately 10 cm skin incision.

Decompression

A complete bilateral L5 facetectomy and laminectomy were performed to decompress the spinal canal. The resected bone was collected and preserved for later use as autologous graft material. Bilateral decompression of the L5 nerve roots was completed prior to pedicle screw placement, allowing direct visualization and protection of the nerve roots during instrumentation.

Instrumentation and Reduction

**Figure 3:**
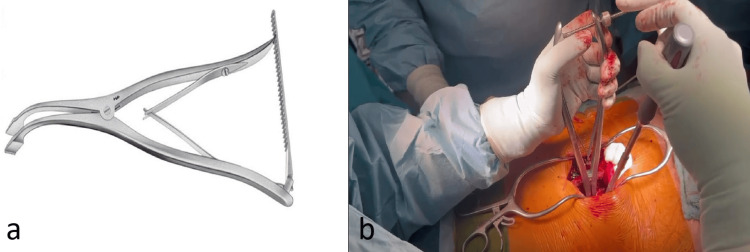
(a) Vertebral body spreader (Wirbelkörperspreizer INGE, Aesculap, Tuttlingen, Germany) for (b) further mobilization of the intervertebral space L5/S1.

The L5/S1 disc space was identified and accessed. Disc material was removed, the endplates were prepared, and the anterior longitudinal ligament was released under fluoroscopic guidance using reamers and rongeurs. To facilitate reduction, the hips were extended (Figure 2B). A vertebral body spreader (Wirbelkörperspreizer INGE, Aesculap, Tuttlingen, Germany) was inserted into the disc space to allow further anterior mobilization (Figure 3). These maneuvers contributed to the correction of the spondylolisthesis and improvement of the slip angle. Mobilization and elevation of the L5 vertebra also optimized the entry point and trajectory for L5 pedicle screw placement.

Pedicle screws (CFX model, DePuy Synthes, Raynham, MA) measuring 5 × 50 mm were inserted into the L5 pedicles under fluoroscopic guidance and direct visualization of the L5 nerve roots. Subsequently, under biplanar fluoroscopy, a cannulated needle was introduced from the caudal posterolateral aspect of S1, traversing the L5/S1 disc space toward the inferior ventral portion of the L5 vertebral body. A K-wire was advanced through the needle, which was then removed. After tapping with a 5 mm tap, 6 × 60 mm cannulated pedicle screws (CFX model) were placed transsacrally.

Cage Placement

Autologous bone graft was packed into the L5/S1 intervertebral space. An expandable lordotic interbody cage (Centrum, Baide Medical, Zhuhai, China), measuring 9 × 23 mm with an expansion range of 10-14 mm, was filled with autologous bone graft and inserted via an ipsilateral transforaminal approach. The cage was positioned between the two transsacral screws. Slightly kyphotic rods were selected and secured bilaterally. Lordotic rods were intentionally avoided, as adequate reduction had already been achieved through disc space mobilization. This strategy minimized the risk of compromising fixation strength in the setting of potentially dysplastic L5 pedicles and avoided redundant reduction maneuvers, which would not have been feasible due to the initial fixation of L5 and S1 with transsacral screws.

Final fluoroscopic imaging confirmed appropriate screw and cage positioning, restoration of disc height, and correction of the slip angle. An additional autologous bone graft was placed posterolaterally over the transverse processes to promote fusion. A wound drain was inserted, and the incision was closed in layers.

Postoperative course

The total operative time was 191 minutes, with an estimated blood loss of 900 ml. One unit of packed red blood cells was transfused postoperatively.

**Figure 4:**
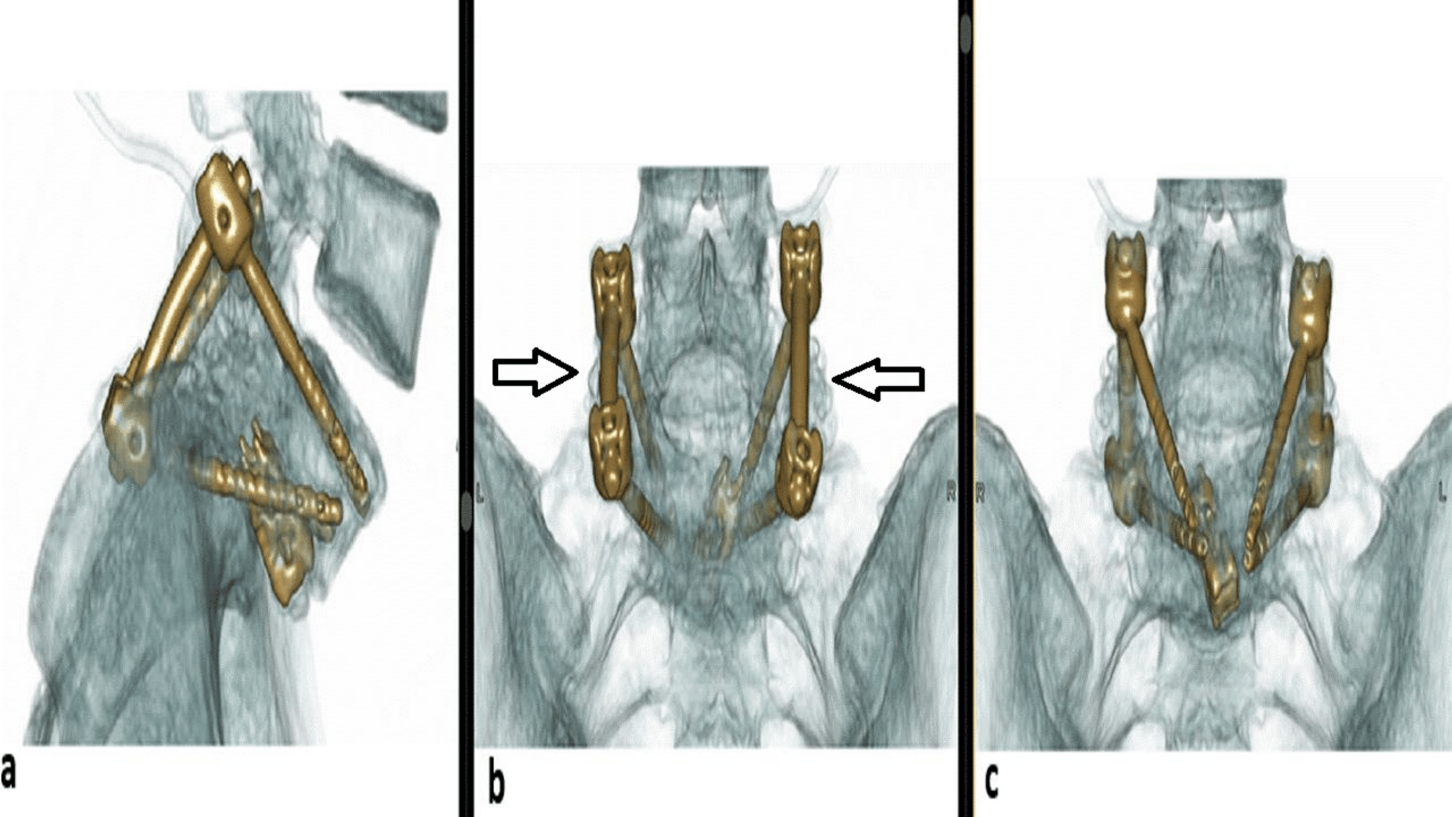
3D CT scan reconstruction showing the positioning of the screws and expandable cage six months postoperatively with solid fusion posterolateral (arrows): (a) lateral, (b) posterior, and (c) anterior view.

The patient was mobilized on postoperative day two with a lumbar orthosis. She was discharged on postoperative day seven. The brace was continued during early recovery for three months, and the patient progressively returned to daily activities. At six months of follow-up, she was pain-free with complete resolution of symptoms and VAS scores of 0/10 for both low back and leg pain. Computed tomography demonstrated solid posterolateral fusion at L5/S1 with no loss of reduction (Figure 4).

## Discussion

There remains a lack of high-quality evidence guiding the surgical treatment of high-grade spondylolisthesis, with most studies classified as level III or IV. Despite numerous described techniques, there is no established consensus, and the optimal management strategy continues to be debated [7,8]. A recent review of the historical evolution and management of high-grade spondylolisthesis identified 12 different surgical techniques [6].

**Figure 5:**
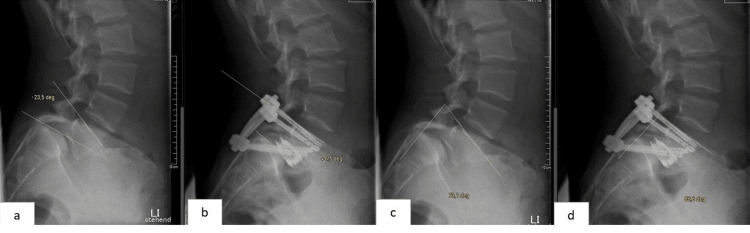
Preoperative and postoperative standing X-ray views showing a 30° reduction of slip angle (a and b) and preoperative and postoperative standing X-ray views showing a 13.5° reduction of lumbosacral angle (c and d).

This technical note introduces a novel combination of transsacral screws with an expandable cage inserted via a TLIF approach for high-grade spondylolisthesis. Each technique is individually accepted for managing high-grade slips, but their combination has the potential to further enhance surgical outcomes [9-13]. The technique simultaneously addresses multiple objectives, including direct decompression of neural elements, partial reduction of high-grade spondylolisthesis, correction of slip angle and lumbosacral kyphosis, and restoration of anterior column support (Figure 5). These combined effects contribute to enhanced biomechanical stability at the lumbosacral junction, improved sagittal alignment, and potentially higher fusion rates. Compared with conventional anterior-posterior fusion techniques, this method avoids anterior exposure and therefore reduces anterior soft tissue disruption and the risk of approach-related complications.

The operative duration was 191 minutes, with an estimated blood loss of 900 ml, necessitating transfusion of one unit of packed red blood cells postoperatively. Although these values are higher than those typically reported for standard single-level lumbar fusion procedures, they remain within the range described for surgical management of high-grade spondylolisthesis, where extensive decompression and reduction are often required [14]. In this context, Faldini et al. reported a mean operative time of 275 ± 65 minutes in their case series, supporting that the operative duration in our case is comparable to previously published experiences for complex high-grade slips [14].

A key advantage of this approach is that it avoids extending instrumentation to L4 or the iliac bones, which can increase surgical morbidity, blood loss, and reduce spinal mobility [5,15]. By limiting the construct to L5 and S1, we preserve adjacent segment integrity while still achieving adequate biomechanical support and fusion, which is particularly relevant in younger patients where long-term motion preservation is a priority. However, Guo et al. (2025) reported an increased risk of adjacent segment spondylolisthesis or instability following L5-S1 fixation compared with L4-S1 fixation in children with high-grade dysplastic spondylolisthesis, though they concluded that these findings do not necessarily justify extending fixation to L4 [16]. Many authors currently extend instrumentation to L4 or higher to provide initial stability, often planning a second surgery to remove upper screws after fusion [17,18]. This staged approach increases morbidity and operative risk.

With our technique, a second surgery can be avoided, as the construct provides immediate mechanical stability through the combined use of transsacral screws and an expandable cage. The expandable cage can be inserted in a collapsed form, reducing the need for excessive distraction, minimizing endplate trauma, and lowering the risk of subsidence and dural tear. It restores interbody height and lordosis more effectively, enhancing anterior column support and overall biomechanical stability immediately postoperatively [19].

The biomechanical rationale for combining transsacral screws with an expandable cage is that transsacral screws act as an internal strut countering shear forces, while the expandable cage restores disc height and maintains endplate contact, achieving load-sharing across the anterior and posterior columns. This synergy may improve fusion rates and early stability compared with either technique alone. Unlike “banana” cages, a straight expandable cage can be safely accommodated between bilateral convergent transsacral screws, allowing maximal mechanical advantage while avoiding excessive manipulation of neural structures.

This technique is particularly suited for young patients with good bone quality, where preservation of adjacent segments and avoidance of iliac fixation are priorities. It may be less suitable in patients with severely osteoporotic bone, extremely narrow disc spaces, or anatomical variations that prevent safe placement of transsacral screws.

While our early experience has been favorable, the use of transsacral screws carries theoretical risks of screw misplacement, sacral fracture, and injury to anterior vascular structures. Additionally, as with all lumbosacral fusion constructs, there remains the potential for adjacent segment disease over time. However, avoiding iliac fixation reduces the risk of hip joint complications associated with spinopelvic instrumentation [20], but long-term follow-up is needed to confirm these benefits.

Limitations

This report describes a single case with limited follow-up and no standardized functional outcome measures. Therefore, generalizability is limited, and long-term outcomes remain uncertain. Additionally, global spinopelvic parameters, including spinal axis-femoral head alignment, were not assessed, which may have implications for adjacent segment balance. Future studies with larger cohorts, biomechanical evaluations, comparative designs, and extended follow-up are required to validate safety, efficacy, functional outcomes, and the impact on spinopelvic alignment.

Despite these limitations, early postoperative results indicate that this technique is technically feasible, safe, and promising in providing spinal stability for complex lumbosacral pathology while potentially reducing morbidity associated with extended instrumentation or staged procedures.

## Conclusions

The presented technique offers a novel and effective alternative for the surgical management of high-grade L5/S1 spondylolisthesis, particularly in young patients. By combining transsacral screw fixation with an expandable interbody cage via a TLIF approach, the method achieves key surgical goals: direct neural decompression, reduction of the olisthesis, restoration of disc height, and correction of lumbosacral alignment. Notably, this construct avoids extension of instrumentation to the L4 vertebra or iliac bones, preserving adjacent segment mobility and potentially reducing long-term complications such as hip arthritis or adjacent segment disease. Early outcomes in the case presented demonstrate successful fusion, pain relief, and restored stability without the need for staged surgery. While existing literature has documented each technique independently, this is the first known published report of their combined use in high-grade spondylolisthesis. The synergistic biomechanical benefits support a structurally robust alternative to more extensive fusion constructs. Long-term follow-up and comparative studies are necessary to validate the durability and safety of this technique, but initial results are promising, suggesting it may fill an important gap in the treatment of complex lumbosacral deformities.
